# Influence of Hypertension on Neurocognitive Domains in Nondemented Parkinson's Disease Patients

**DOI:** 10.1155/2014/507529

**Published:** 2014-01-23

**Authors:** Jacob D. Jones, Charles Jacobson, Martina Murphy, Catherine Price, Michael S. Okun, Dawn Bowers

**Affiliations:** ^1^Department of Clinical and Health Psychology, McKnight Brain Institute, University of Florida, 101 South Newell Drive, Gainesville, FL 32601, USA; ^2^Center for Movement Disorders and Neurorestoration, McKnight Brain Institute, University of Florida, 3450 Hull Road Gainesville, FL 32607, USA; ^3^Department of Internal Medicine, McKnight Brain Institute, University of Florida, P.O. Box 100277, Gainesville, FL 32610-0277, USA; ^4^Department of Neurology, McKnight Brain Institute, University of Florida, HSC, P.O. Box 100236, Gainesville, FL 32610, USA

## Abstract

*Objective*. Health comorbidities, particularly cardiovascular risk factors, are well known to pose risks for cognitive decline in older adults. To date, little attention has focused on the impact of these comorbidities on Parkinson's disease (PD). This study examined the prevalence and contribution of comorbidities on cognitive status in PD patients, above and beyond the effects of disease severity. *Methods*. A cross sectional design was used, including neuropsychological data on 341 PD patients without severe cognitive decline. Comorbidity data were collected via medical chart review. Data were analyzed using a series of multiple hierarchical regressions, controlling for PD-related disease variables. *Results*. Overall sample characteristics are 69% male, disease duration 9.7 years, Unified Parkinson's Disease Rating Scale 26.4, and age 64.7 years. Hypercholesterolemia (41.6%), hypertension (38.1%), and hypotension (30.2%) were the most reported comorbidities. The presence of hypertension significantly contributed to domains of executive function and verbal memory. The cooccurrence of orthostatic hypotension moderated the relationship between hypertension and executive function. *Conclusions*. This study on a large cohort of PD patients provides evidence for a detrimental influence of health comorbidities, particularly hypertension, on cognitive domains that have traditionally been conceptualized as being frontally and/or temporally mediated.

## 1. Introduction

The overall goal of this proof of concept study was to learn whether cardiovascular risk factors, like hypertension and diabetes, might negatively influence cognitive status in Parkinson's disease, similar to that observed in normal elderly. Parkinson's disease (PD) is a complex multisystem disorder characterized by motor, cognitive, and mood-motivational changes [1, 2]. Particularly, insidious are cognitive changes. When initially diagnosed, 5–20% of PD patients show signs of cognitive difficulties and up to 80% become demented after 15–20 years [3, 4]. Typical cognitive changes include slowed processing (bradyphrenia), increased forgetfulness, and difficulty with multitasking and working memory. Cognitive changes can occur early in the disease course, worsen with disease progression, and detrimentally affect quality of life and survival [3, 5]. From a neural systems perspective, PD-related cognitive decline has been attributed to deregulation of dopamine-mediated frontal-striatal circuitry and is further complicated by cholinergic changes [6, 7].

Over the past decade, mounting evidence has pointed to the detrimental effects of vascular risk factors on cognitive decline in “normal” older adults [8]. Hypertension and diabetes have emerged as leading risk factors for declines in attention, memory, processing speed, and executive functions [8, 9]. One proposed mechanism accounting for cardiovascular-linked cognitive changes is small vessel disease, seen as leukoaraiosis (LA) on magnetic resonance imaging (MRI) [10]. In addition to LA, diabetes is related to metabolic disruption, which preferentially impacts hippocampal regions involved in memory recall [11].

By contrast, few studies have examined the influence of cooccurring health conditions (particularly hypertension) on cognitive status of Parkinson patients. In part, this relates to lower occurrence of hypertension in PD, secondary to antihypertensive effects of levodopa medications. Of those studies investigating the relationship between vascular risk factors and cognition in PD, findings have been mixed. One group of studies (*N* = 3) has used “dementia status” as an outcome and has found no relationship between presence of vascular risk factors and a diagnosis of dementia in Parkinson's disease [12–14]. A limitation of this approach is that categorical classification of yes-no dementia status may be less sensitive to changes than a parametric approach. In contrast, a recent study using data from a cohort of almost 2000 PD patients from the National Parkinson Quality Improvement Initiative (NPQII) found that diabetes and “heart/vascular” disease exerted a small but detrimental relationship on two simple measures of cognition (animal fluency and a 5-word recall memory task) [15]. Due to the nature of the NPQII data, the study was unable to isolate the effects of hypertension, eliminate individuals with “dementia”, or examine a range of cognitive domains that are associated with PD decline. The inconsistencies between the latter and prior studies leave open the possibility that while comorbidities may not be directly predictive of dementia status, they may contribute to cognitive changes that predate dementia onset.

The current study examined the effect of a wide range of health comorbidities across distinct neurocognitive domains in patients with Parkinson's disease. Our working hypothesis was that the occurrence of cardiovascular comorbidities would exert detrimental effects on executive function, delayed memory, and processing speed, similar to that observed in normal elderly. To test this hypothesis, we identified a convenience sample of Parkinson patients who had undergone comprehensive neuropsychological evaluation and scored in the unimpaired range on the Dementia Rating Scale-II (DRS-II; i.e., >5th %Ile) [16]. We then examined the contribution of cooccurring health conditions obtained from medical chart review to specific neurocognitive domains. We specifically predicted that hypertension, often associated with frontal-subcortical deregulation, would be associated with worse cognitive performance across executive, working memory and processing speed domains. We additionally predicted that comorbidities related to impaired glucose metabolism (diabetes) and hypoxic events (i.e., acute cardiac events) would contribute to worse delayed memory recall associated with medial temporal lobe memory systems. Because PD itself induces frontal-executive decline, particularly with disease progression, we statistically controlled for disease severity in order to identify any “added” contribution of cooccurring health conditions on cognition.

## 2. Methods

### 2.1. Design

A cross-sectional design was used and included a convenience sample of patients with idiopathic Parkinson's disease who had undergone a detailed neuropsychological assessment as part of a workup through the University of Florida (UF), Center for Movement Disorders and Neurorestoration. All patients had a diagnosis of idiopathic PD, per UK Brain Bank criteria, by a movement disorder neurologist [17]. The study was approved by the Institutional Review Board (IRB) at the University of Florida.

### 2.2. Participants

Participants included 471 patients with idiopathic PD, seen between January 2006 and September 2010. Participants had undergone neuropsychological evaluation and had information pertaining demographics, medications (including levodopa equivalence dose), health comorbidities, and PD disease severity (i.e., “one medication” scores from the motor exam of the Unified Parkinson's Disease Rating Scale; UPDRS, Part III) [18, 19]. Exclusion criteria included past brain surgery (e.g., deep brain stimulation), severe psychiatric disturbance (schizophrenia, current major depression episode), severe sensory defects (blindness, deafness), and impaired scores (<5th percentile) on the Dementia Rating Scale-II (DRS-II) [16]. Of the total 471 patients reviewed, 130 individuals were excluded as follows: 65 due to low scores on the DRS-II, 42 due to previous brain surgery, 17 for missing UPDRS motor scores, and 6 for missing information regarding disease duration. The final sample included 341 individuals.

### 2.3. Neurocognitive Measures

Table 1 shows the specific cognitive measures that were given as part of the neuropsychological evaluation. Tests were grouped into five rationally derived domains following the approach of Sheline et al. [20] and included attention/working memory, delayed episodic memory (verbal), language, executive skills, and processing speed. With the exception of  “language,” all cognitive domains included at least two tests. Scores from each measure were normed from test-specific manuals or previously published norms and then converted to *Z*-scores [21]. Composite scores for each domain were calculated by averaging the normed *Z*-scores within a domain. The advantages of using composite scores include better reliability with multiple measures per construct of interest [16]. In the event that a patient had multiple neuropsychological evaluations, only data from the initial exam was used.

### 2.4. Comorbidities

Health comorbidities were documented by review of medical records at UF-Shands during the year prior to the neuropsychological exam. A listing of over 50 health comorbidities was initially compiled based on previous studies (i.e., Framingham Heart Study and Charlson Comorbidity Index) and reviewed by physicians from neurology and internal medicine [22, 23]. Comorbidities were coded dichotomously for presence or absence by a single rater. Prior to the chart review, adequate reliability was established with two additional raters across 10 charts (interrater range: *r* = 0.97–0.98). Comorbidities were considered present if (1) the physician noted its occurrence in a medical note, or (2) the patient was prescribed medication pertaining to its presence (e.g., levothyroxine for hypothyroidism). Blood pressure readings were retrieved from medical records. In accord with past studies investigating cognition and hypertension, individuals with a systolic blood pressure ≥160 mmHg, or those on antihypertensive medication were considered as hypertensive [24]. Because some specific health conditions occurred relatively infrequently, they were combined into three broad categories; cardiac, respiratory, and neurologic. The *cardiac* category included heart attack, angina, atrial fibrillation, arrhythmia, heart murmur, congestive heart failure, peripheral vascular disease, or any cardiac surgery. The *respiratory* category included chronic obstructive pulmonary disease, pulmonary embolism, emphysema, and asthma. The *neurologic* system comorbidity included stroke, TIA, head injury, and peripheral neuropathy.

## 3. Results

Characteristics of the 341 Parkinson samples are shown in Table 2. Overall, the PD patients were in their mid-60′s, well educated (i.e., approximately 3 years of college), and predominantly Caucasian (94%) and male (69%). In terms of disease characteristics, the majority of PD patients were tremor predominant (82.7%) with disease duration ranging from 1 to 33 (mean of 10 years), and a mean “on medication” UPDSR motor score of 26.4. Approximately 25% were on antianxiety medications and approximately 40% on antidepressants. The mean DRS-II score was 137.3 with a standard deviation (SD) of 5. As a group, performance was lowest on the processing speed composite score (mean = −0.74), followed by executive functioning (mean = −0.56) and delayed verbal memory (mean = −0.4). Regarding mild cognitive impairment (MCI) subtypes, the guidelines of the Movement Disorders Society taskforce (25) were used for classification into cognitive subgroups. A clinical cutoff criteria of 1.5 SD below the normative mean was considered “impaired.” Following the MDS guidelines, 180 PD participants (52.8%) were classified as cognitively intact, 103 (30.2%) as multidomain amnestic MCI, 57 (16.7) as nonamnestic MCI, and 1 (0.3%) as single domain amnestic MCI.

**Figure 1:**
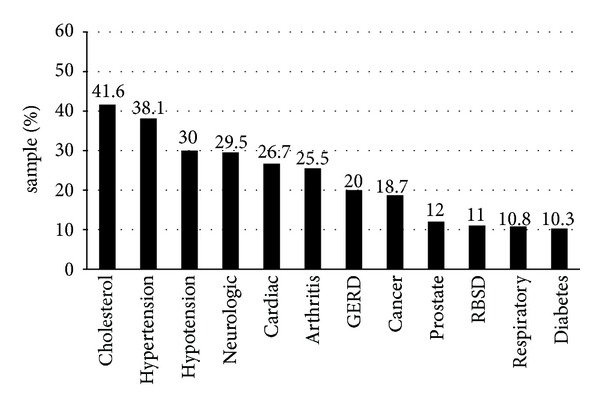
Prevalence of comorbidities in a convenience sample of 341 idiopathic Parkinson patients (*N* = 341). Shown is the percentage of Parkinson patients with various comorbidities based on medical chart review; only comorbidities occurring in at least 10% of the sample are shown. GERD = gastroesophageal reflux disease; RBSD = rapid Eye Movement (REM) behavior sleep disorder. None have deep brain stimulation.

Figure 1 shows the distribution of 12 comorbidities that occurred in at least 10% of the sample. The three most common comorbidities were cholesterolemia (41.6%), hypertension (38.1%), and orthostatic hypotension (30%).

### 3.1. Relationship between Cognition and Cardiovascular Risk Factors

To assess the “unique” effect of health comorbidities (including cardiovascular risk factors) on each of the five cognitive domains, a series of hierarchical regression analyses were conducted. The first block of predictors in each regression included indices of PD severity (UPDRS motor scores, disease duration, and LED), whereas the second block of predictors included subsets of health comorbidities (i.e., hypertension, diabetes, cancer, and arthritis). This approach enabled one to determine the relation between health conditions and cognition, while statistically controlling for the influence of PD disease severity. This is important due to well known association between disease severity itself and cognitive decline [3]. For each regression, the cognitive domain composite score served as the dependent variable. Of note, only health comorbidities that involved at least 10% of the sample were included for the purpose of stability. For all regression analyses, collinearity diagnostics (tolerance > 0.2 and variation inflation factor < 5) and normality (skewness and kurtosis values < 1) were appropriate. Post hoc tests utilized least significant difference tests [26].

As shown in Table 3, the *overall* regression models were significant for three of the cognitive domains: executive function, delayed verbal memory, and processing speed. Because the regression models for the two remaining cognitive domains (language and working memory) were not significant, they were not examined further. For all three “significant” cognitive domains (executive, verbal memory, and processing speed), Parkinson's disease severity exerted a significant effect on cognition. Specifically, higher UPDRS motor scores and longer disease duration were associated with worse composite scores on executive, delayed memory, and processing speed. As a whole, the addition of “health comorbidities in general” was not significant for any cognitive domain. However, significant effects were present for specific cardiovascular risk factors, namely hypertension and orthostatic hypotension. A diagnosis of hypertension (even controlled hypertension) was associated with worse executive function and delayed verbal memory scores. These effects were significant, but nevertheless small. Unexpectedly, the diagnosis of orthostatic hypotension (OH) was associated with better scores on tasks of executive function. Processing speed scores were not significantly related to any comorbidity.

Although cognitive data was normed on age and education, subsequent analyses controlling for age and education confirmed the above findings and reinforced that the previous analyses were not confounded by differences in age or education. Multinominal regression analyses examined differences in health comorbidities among cognitive subtypes as the outcome (i.e., cognitively intact, amnestic and nonamnestic MCI groups). Results revealed no significant differences in the prevalence of comorbidities.

### 3.2. Hypertension-Hypotension Subgroups

**Figure 2:**
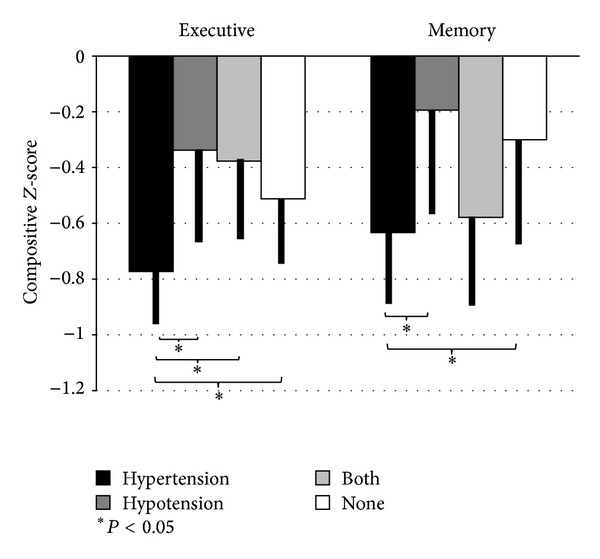
Executive and verbal memory composite scores in four blood pressure groups; hypertensive, hypotension, cooccurring hypertension and hypotension (both), and normotensive (none). Figure depicts mean composite *Z*-scores for executive function and delayed verbal memory across each of the blood pressure groups. For *executive function*, Parkinson's patients with hypertension alone (*X* = −0.781, SD = 0.769) performed significantly worse than those with hypotension alone (*X* = −0.321, SD = 0.769, *t* = −2.87, *P* = 0.004), combined hypertension-hypotension (*X* = −0.437, SD = 0.739, *t* = −2.50, *P* = 0.013), and normotensive/none (*X* = −0.495, SD = 0.898, *t* = −2.21, *P* = 0.028). For *delayed verbal memory*, Parkinson's patients with hypertension alone (*X* = −0.633, SD = 1.068) performed significantly worse than those with hypotension alone (*X* = −0.194, SD = 1.098, *t* = −2.45, *P* = 0.015) and the normotensive/none group (*X* = −0.301, SD = 1.013, *t* = −2.23, *P* = 0.026). There were no differences in memory between the hypertension and the combined hypertension-hypotension groups.

Hypertension and orthostatic hypotension (OH) often cooccur in individuals with PD. Consequently, additional analyses were conducted to more closely examine the separate and combined influence of hypertension and orthostatic hypotension on executive function and memory. By way of background OH refers to a drop in blood pressure due to postural changes, such as standing, and can induce dizziness, light-headedness, or falls [24]. Orthostatic hypotension is a common complication in PD due to sympathetic denervation and norepinephrine degeneration in the locus coeruleus [27]. Paradoxically, OH can cooccur with hypertension, since the mechanisms underlying each are quite different. For the current analyses, patients were assigned to one of four groups: those with orthostatic hypotension alone (OH, *N* = 55), those with hypertension alone (HTN, *N* = 82), those with cooccurring OH and hypertension (both, *N* = 48), and individuals with neither comorbidity (normotensive/none, *N* = 156). Separate analyses of covariance (ANCOVA) were conducted using group (OH, HTN, both, normotensive/none) as the between-subjects factor and covarying for disease severity (UPDRS motor score, disease duration, LED). Executive and memory composite scores were the dependent variables in each analysis. Results of these ANCOVAs revealed a significant main effect of group for both *executive function* (F(6,328) = 3.568, *P* = 0.015) and *delayed verbal memory* (F(6,328) = 2.680, *P* = 0.047). Post hoc tests using least significant difference [26] revealed that the Hypertension group performed worse than all other groups for executive functioning (all *P* values <0.05), with no significant differences among the OH, both, or normotensive groups (Figure 2). For delayed memory, the hypertension group performed worse than the OH (*P* = 0.015) and the normotensive groups (*P* = 0.026), but not the combined/both group.

### 3.3. Pulse Pressure

Because pulse pressure has been described as a more sensitive measure of cardiovascular risk than either systolic or diastolic pressure alone [23], we performed additional exploratory analyses examining the relationship between pulse pressure, cognition, and PD disease severity (UPDRS motor scores). Pulse pressure was computed from blood pressure readings reported in the medical charts, following standard guidelines, by subtracting diastolic from systolic blood pressure values. Again, a series of multiple hierarchical regression analyses were conducted using cognitive domain scores as the outcome variable in each. To control for disease severity, PD severity measures (UPDRS, symptom duration, LED) were included in the first regression block. The second block included pulse pressure and hypertensive medication (recorded as a dichotomous variable for presence). A third block included a residualized pulse pressure by UPDRS interaction term. The latter enabled us to examine whether PD severity and vascular risk interacted to influence cognitive outcomes. A similar approach has previously been suggested by studies investigating the relationship between white matter changes (leukoaraiosis) and motor functioning in patients with PD [25].

**Figure 3:**
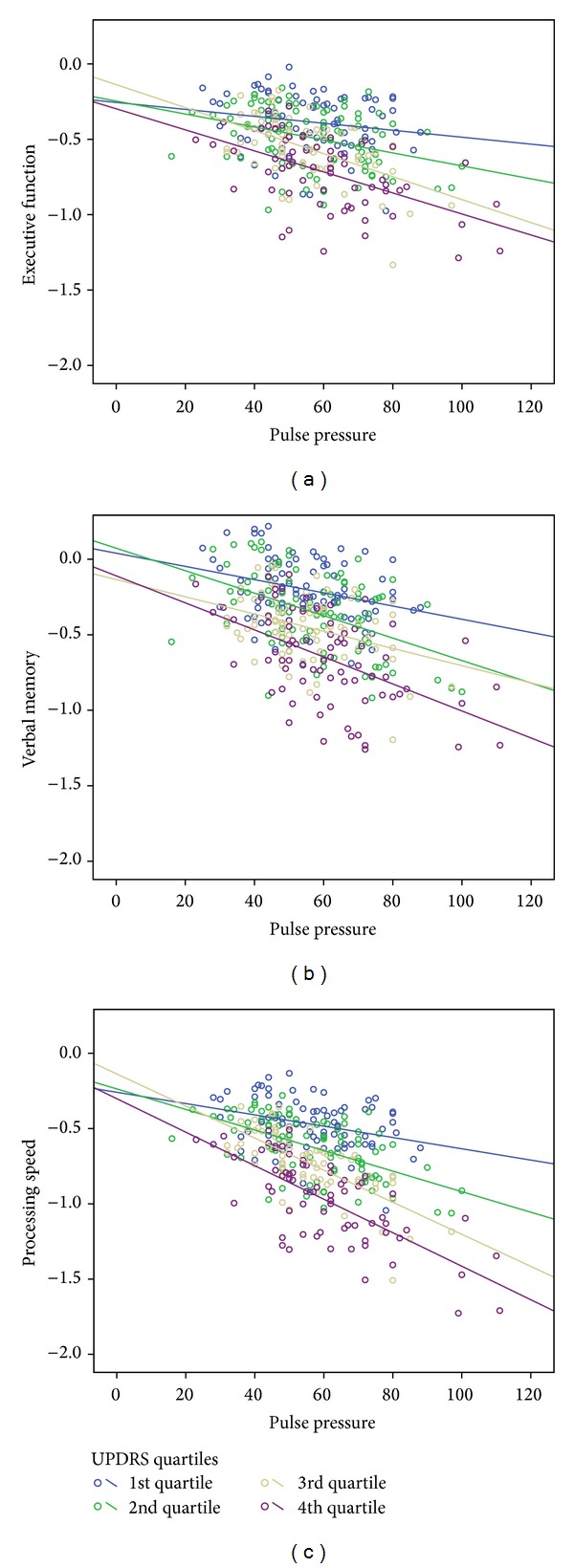
Pulse pressure *X* UPDRS interaction for 3 cognitive domains. Figure depicts linear relationship between pulse pressure and executive function, delayed verbal memory, and processing speed for each Unified Parkinson's Disease Rating Scale Part III (UPDRS) quartile (higher quartile means more severe motor symptoms).

Results of the overall regression model was significant for executive functioning, delayed verbal memory, and processing speed, but not for working memory or language (Table 4). The PD severity block was significant across all three domains; whereas the second block (pulse pressure and hypertensive medication) was not significant for any cognitive domain. However, there was a *significant interaction between UPDRS motor scores and pulse pressure* for executive function, delayed verbal memory, and processing speed. Specifically, higher pulse pressure values were significantly related to worse cognitive performance as UPDRS motor scores increased in severity (Figure 3). This pattern was observed for executive function, delayed verbal memory, and processing speed. Taken together, this finding suggests that worsening vascular integrity (as reflected by pulse-pressure) interacts with PD-indices of disease severity to exert increasingly detrimental effects on executive function, memory, and processing speed.

## 4. Discussion

This retrospective study with 341 nondemented Parkinson patients resulted in three important findings. First, while disease severity is a potent indicator of cognitive performance in PD, we found that hypertension, a common age-related comorbidity that occurred in almost 40% of our sample, exerted a small but independent negative influence on composite measures of executive function and delayed verbal memory. This finding aligns itself with a large literature in normal elderly showing the detrimental impact of hypertension on cognition, particularly executive functions [28] and is consistent with recent observations from a large multicenter Parkinson cohort [15]. Interestingly, we did not find support for our hypothesis of diabetes or acute cardiac events relating to memory functioning. Previous findings in non-PD elders have shown both comorbidities to be related medial temporal lobe functioning [11]. This discrepancy may reflect our small sample size of diabetics (*N* = 35), and the heterogeneity of comorbidities included under the “cardiac system” variable.

Our finding that hypertension in PD was associated with worse executive function and delayed memory is in line with a large literature in older adults documenting negative influence of cardiovascular risk factors. However, our results contrast with a small group of studies that failed to find a relationship between cardiovascular risk and transition to dementia in Parkinson patient [12–14]. We suspect that there are several factors that account for the different findings. The first relates to our use of parametric indices of cognition rather than a categorical classification of dementia presence used in previous PD students. The former approach is a more sensitive metric for detecting cognitive change or differences. Second, our sample size was considerably larger than previous PD studies and this may be critical given the relatively small effect sizes we observed. Additionally, previous studies of cognitive status have shown executive functions to be an insensitive marker of progression to dementia [7]. Thus, while hypertension may relate to executive dysfunction, this cognitive profile may not differentiate dementia from nondemented PD patients. However, this view is debatable and has not been established [29].

To be clear, the focus of the present study was not to identify those with and without dementia, but to learn whether hypertension and other comorbidities might contribute to the cognitive profile of Parkinson patients without severe cognitive impairment based on a general cognitive screener (DRS-II). Evidence from this study suggests that hypertension does indeed make a small, but significant contribution, particularly to executive function and delayed memory recall. Of note, the occurrence of hypertension in our PD sample is less than that often observed in several previous reports describing hypertension to occur in 53–70% of individuals above the age of 65 [24]. Possible explanations for this discrepancy includes a protective effect against hypertension secondary to levodopa use [30] as well as the fact that this study includes individuals younger than 65 years old.

The second major finding was an unexpected relationship between executive function and orthostatic hypotension, which occurred in approximately 30% of our sample. Our findings suggested a possible role of orthostatic hypotension (OH) in moderating the relationship between hypertension and executive function. In brief, individuals with cooccurring hypertension and OH were on par with individuals without hypertension on executive functioning measures. This phenomenon has not been previously well documented or established and requires further validation. The relationship between OH and cognition in the normal elderly has revealed mixed results with some studies failing to find a relationship between cognitive outcomes and OH [31]. However, one study found that the combination of both hypertension and orthostatic hypotension served as a protective factor [32]. Specifically, Yap and others found that among 2,321 older adults, those with both OH and high blood pressure (>140 systolic or >90 diastolic) were less likely to have impaired scores on a broad measure of cognition (MMSE), compared to individuals without OH, and those with OH and low blood pressure. The mechanism for this effect has not been thoroughly examined but may relate a reduction in mean arterial pressure associated with OH, allowing for cerebral blood perfusion to stay in the range of autoregulation [33]. Within PD, a recent study showed that PD patients with circulatory abnormalities (including OH and supine hypertension) were more likely to meet criteria for dementia [34]. Additionally, they found that tests of episodic memory, but not executive functions, were significantly related to decreases in blood pressure during a head-up-tilt test. Such a finding may suggest that dysautonomia may be related to cognitive changes occurring later in the disease stage, rather than frontal-executive changes occurring earlier.

Third, this study showed a unique finding with pulse pressure, whereby, higher pulse pressure interacted with PD severity to exert increasingly detrimental effects on executive function, processing speed, and memory. In other words, the relationship between cognition and cardiovascular risk may be moderated by PD severity. We are unaware of previous studies examining the effect of pulse pressure on cognition in Parkinson patients. There are several ways to conceptualize this relationship. On one hand, worsening pulse pressure may be another index of PD progression vis a vis worsening autonomic dysfunction and effects on blood pressure. Alternatively, this relationship may be independent of autonomic dysfunction. In either case, white matter changes might represent downstream effect of this variability. A growing number of studies have found that the relationship between LA and cognition varies depending on PD severity. In fact, studies of Parkinson patients in more advanced stages have shown LA to be related to cognition, whereas studies of newly diagnosed PD patients failed to replicate this finding [12–14, 35], suggesting that vascular pathology may interact with PD pathology. This idea of an interaction between vascular pathology and PD pathology has also been explored in its relationship to non-cognitive symptoms of PD. In terms of motor complications in PD, higher LA burden has been associated with worsening motor functioning, including postural/gait difficulty, bradykinesia, and rigidity [36].

There are several limitations to this study. First, the design was cross-sectional and used a convenience sample of patients which may limit generalizability. Next, comorbidity measures depended on accurate documentation by physicians in the patients' medical charts. While the validity of this method is unknown, we believe the most noteworthy comorbidities were likely noted and recorded as part of the patient's medical record. Lastly, other than blood pressure, there were no direct measures of comorbidity, and we were unable to quantify severity or duration of various health conditions. Concerning blood pressure, data consisted of one measurement, rather than multiple blood pressure measurements which is more advantageous.

## 5. Conclusion

The current study adds to the literature by suggesting that hypertension may be related to subtle executive and memory deficits in Parkinson patients, above and beyond that conferred by the disease process itself. The effect is likely small, though it does parallel that observed in non-PD older adults, and may become more salient with disease progression. What is particularly unique in Parkinson's disease is a juxtaposition of factors that are protective versus those that increase cardiovascular risk. Protective factors include use of levodopa medications that decrease blood pressure, whereas autonomic dysfunction, use of dopamine agonists (related to cardiac valvulopathy), and general physical deconditioning serve to increase cardiovascular risk [31]. Even with this unique confluence, the present study provides preliminary evidence that hypertension may be related to subtle cognitive deficits in Parkinson patients, similar to that of older adults, and may amplify executive and memory difficulties. Future studies should examine possible mechanisms, such as LA and the impact that subtle vascular-related cognitive deficits have on quality of life and mood. Improving cardiovascular health, prior to dementia onset, should be further investigated as a means of moderating cognitive changes. Due to high risk of cognitive decline/dementia, and the degenerative nature of PD, a small minimization in risk of cognitive decline may prove to be beneficial.

## Figures and Tables

**Table 1:** Cognitive domains.

Domain	Measures and brief description
Working memory	Digit Span: measure of auditory attention and working memory from the WAIS-III, with both forward and backwards components, dv = forward span, and backwards span scores

Episodic memory	Hopkins Verbal Learning Test-*R*, Form 1 (HVLT-R): 12 item word list learning task with 20 minute delay; dv = # items recalled after 20′ delay
	Logical Memory Stories II (WMS-III): story memory measure, dv = # units recalled after 30 min delay

Language	Boston Naming Test (BNT): confrontation naming of line drawings of objects/animals; dv = total items correctly named

Executive function	Trail Making Test, Part B (TMT-B): speeded alternating search of letters and numbers (set-shifting), dv = total time
	Stroop Color-Word Test (Golden version): measure of cognitive inhibition of over learned automatic reading response; dv = number items within 45 sec on color-word trial
	Controlled Oral Word Association Test (COWA): a letter fluency task involving production of words beginning with target letters during 60 second trials; dv = number words produced

Processing speed	Stroop Single Word Reading (golden version): timed reading of single words denoting red, green, or blue; dv = number words read in 45′′
	Trail Making Test, Part A (TMT-A): speeded search of letters displayed on a page; dv = total time

Note: WMS-III: Wechsler Memory Scale-Third Edition; WAIS-III: Wechsler Adult Intelligence Scale-Third Edition; dv: dependent variable. References for individual tests are in Lezak et al., (2012) [16].

**Table 2:** Sample characteristics.

*N* = 304 Parkinson's disease patients 88% caucasian	Mean	Standard deviation	Range
Age (years)	64.7	10	30–90
Years of education	15.0	3	7–20
Years with symptoms	9.7	6	1–33
UPDRS motor score, on medication	26.3	10	7–54
Percent tremor predominant	82		
Levodopa equivalency dose	771	532	0–2950
Dementia Rating Scale-II total score	137.2	5	119–148
Neurocognitive domain *Z*-scores*			
Executive composite score	−0.6	0.9	−2.9–1.9
Verbal memory composite score	−0.4	1.1	−3.7–1.8
Processing speed composite score	−0.7	0.9	−3.2–1.9
Language composite score	0.2	1.2	−4.0–3.2
Working memory composite score	0.2	0.8	−1.9–2.7
Mild cognitive impairment (MCI) subtypes			
Percent amnestic single-domain MCI	0.3		
Percent multidomain amnestic MCI	30.2		
Percent nonamnestic MCI	16.7		
Percent cognitively intact	52.8		

*Z-scores based on normative data from test manuals and published norms: normative mean = 0, standard deviation = 1; mild cognitive impairment was based on Movement Disorder Society criteria and utilized a 1.5 SD below the mean cut value. [25] UPDRS: Unified Parkinson's Disease Rating Scale.

**Table 3:** Comorbidities and cognitive domains: summary of results of hierarchical multiple regression analyses.

	*F*Δ	*R* ^2^Δ	Beta	Sig
*Executive function *				
Final model	3.38	0.138		<0.001
PD severity block	10.247	0.091		<0.001
Duration			−0.156	<0.001
Motor UPDRS			−0.246	<0.001
Comorbidity block	1.460	0.047		0.146
Hypertension			−0.127	0.041
Hypotension			0.134	0.019

*Delayed verbal memory *				
Final model	3.39	0.122		<0.001
PD severity block	9.383	0.078		<0.001
Duration			−0.188	<0.001
Motor UPDRS			−0.227	<0.001
Comorbidity block	1.433	0.043		0.157
Hypertension			−0.139	0.021

*Processing speed *				
Final model	3.977	0.154		<0.001
PD Severity block	15.647	0.129		<0.001
Duration			−0.139	0.019
Motor UPDRS			−0.315	<0.001
Comorbidity block	1.433	0.043		0.157

Only significant predictors are shown. PP: pulse pressure; UPDRS: Unified Parkinson's Disease Rating Scale—Part III.

**Table 4:** Pulse pressure and cognitive domains: summary of results of hierarchical multiple regression analyses.

	*F*Δ	*R* ^2^Δ	Beta	Sig
*Executive function *				
Final model	6.297	0.112		<0.001
Duration			−0.195	<0.001
Motor UPDRS			−0.218	<0.001
PP × UPDRS			−0.113	0.043
*Delayed verbal memory *				
Final model	7.603	0.110		<0.001
Duration			−0.127	0.027
Motor UPDRS			−0.213	<0.001
PP × UPDRS			−0.138	0.010
*Processing speed *				
Final model	6.403	0.152		<0.001
Duration			−0.157	0.006
Motor UPDRS			−0.305	<0.001
PP × UPDRS			−0.128	0.017

Only significant predictors are shown. PP: pulse pressure; UPDRS: Unified Parkinson's Disease Rating Scale—Part III.
